# The Lying Patent Medicine Man and the False Certifier*From Proceedings of the American Medical Edit rs’ Association, St. Louis, Mo., June, 1910.

**Published:** 1911-06

**Authors:** Charles H. Hughes

**Affiliations:** Editor Alienist and Neurologist, St. Louis, Mo.


### Selected.

### The Lying Patent Medicine Man and the False Certifier.*

BY CHARLES H. HUGHES, M. D.,
Editor Alienist and Neurologist, St. Louis, Mo.

The prototypes of Blaise in Harrison Ainsworth's Old St. Paul's, tasting the patent medicines each in turn, and each equally efficacious, from day by day warding off the devastating London plague, are many in our day, and the patent medicine liars and some of our proprietary prevaricators are even more numerous and vigorous enough in powers of misstatement, to put it mildly, to paralyze Ananias. These modern false portrayers of the miraculous merits of their medicines would have put the latter gentleman and his later frater, Munchausen, to blush, and prevented their appearance in history, sacred or profane, or in romantic story as unique liars.

But since Ananias and Sapphira were stricken the liar has never lacked an audience, notwithstanding credulity is as diffusively spread among human minds as the propensity to false-

*From Proceedings of the American Medical Edit rs' Association, St. Louis, Mo., June, 1910.

hood, and proficiency therein, especially where there is motive of gain behind the tail, is especially in evidence in the average patent medicine and proprietary medicine romances of cure.

Back in the days of my youth was the "retired clergyman," "whose sands of life had nearly run out," who, before taking his necessary, old-age-impelled final departure to the land of pure delight, where saints immortal dwell, would bestow the blessing of health to every consumptive who would consent to receive his free prescription and get well, so that there might be no more tuberculosis for him to contemplate from his destined blissful abode in the sanitarium of pious tuberculosis cure saints in heaven.

The prescription of the pious philanthropic ecclesiastic, who sent out his charitable proclamation from the Bible house of New York, consisted essentially of extract of blogetti, if any of you can guess what that is, and which no pharmacist could fill. The prescription had, of course, to be sent to the pious, and the world sympathizing author of the proclamation and prescription to be properly dispensed, and, being exceedingly expensive, this free treatment had to be paid for accordingly as often as filled.

Well, it was supposed that this self-proclaimed revered good Samaritan went to his final reward, taking the extract of blogetti with him, and the consumptives continued to follow as they had preceded him to that bourn whence no traveler returns, and we have some yet among us. His name was given to me as Blodgett. He was a little man, but a big liar. It is a pity he had to die before be got to be a multi-millionaire, for the newspapers did all they could for him. This same consumption cure, after disappearing for a while from the heavenly horizon of quack therapeutics, reappeared, like Halley's comet, as the discovery of Rev. Edward A. Wilson, a mythical theologian, who was unfortunately restored to health and suffering from a philanthropic gold-grabbing palmer eczema, was anxious while scratching for a living to send some medicine free of charge to all sufferers from consumption, asthma, bronchitis, catarrh and the usual quack et ceteras, and, strange to say, his marvelous medicine was the same extract of blogetti which his pious predecessor failed to take with him through St. Peter's heavenly gate. This Blogetti business lately got into the mails, along with oxydonor diaduction and the Ohio Soluble Sulphur. Uncle Sam got the fakers.

Next we may note the Balm of Gilead man, who answers the Biblical question, Is there no balm there? by saying, here you are, fifty cents per bottle, please; larger ones, one dollar. Then comes the wonderful purgatory pill, warranted to clean you out from pyloral to anal exits, pocketbook included, with little pleasant pellets and big results, and Peruna also.

Then there was the great Fluid Extract of Buchu man, who reduced all disease as certain as others are now doing, to wrong working kidneys, which Buchu would make work right, and all would be well then.

Well, it is easy to persuade the average man who may not be living right that something is wrong with these emunctories, even when they do not know there is anything the matter, and induce them to take a safe kidney cure. People have disease of the kidneys and don't know, they say, till they try a safe cure. Well, this Buchu man had his boldhelm buchu painted in blazing letters around the world, even, it is said, from the Gulf of Mexico to the banks of the Nile, and on the Pyramids of Egypt. Everybody was taking buchu tea except me. And the newspapers helped all they could. They and the fools made him rich, and he went to the lunatic asylum with money paresis, which ended in further reckless money ventures and failure. Where is that Buchu man now? Gone—where the woodbine twineth and the kidneys trouble no more.

Then there was Walker's Vinegar Bitters, which contained no alcohol, and pleased the supremely radical temperance people, who thought apple cider ought to be excluded by law from mince pie, so well that they all toned up on it, and were moved to recommend it and move their bowels with it. This wonderful bitters, which was to supplant Hostetter's kept at every bar, and all others, with a drop of alcohol in them, was said to contain a rare Mexican plant, that got its name from Jalapa, Mexico. You can guess how rare that plant is. Well, the newspapers helped him, but he had to die, and Vinegar Bitters disappeared from signboards, newspapers, almanacs and the minds of the people. Then comes Malty's Duff Dope, that all old people prolong their lives on, suggested for all who would "live long and prosper." If only old Methuselah had known this wonderful fact, and had the price, he would have been living yet.

Then there were the various brands and compounds of cod liver oil, warranted to cure consumption, to the deception and impoverishment of thousands who could not afford the high price for the nutrient qualities of this oil, when butter was, in other days especially, a cheaper article than now, and as good as a substitute. Now it is advertised as good for neurasthenia and that tired feeling. Every patent medicine now is good for that tired feeling and neurasthenia, as everything used to be good for liver complaint. Now, about all that is advertised as good for the liver is Carter's Little Liver Pills, proclaimed in big letters on the sides and roofs of all the barns in the country.

A few decades ago Osgood's India Chologogue—India, mind you—it would be no good if advertised as only an American bile persuader and liver tickler. Every farm house had it. It was used to kill chills. The malarial plasmodium was not then known. But this India chologogue has worked itself out of public confidence, because the proprietor is not, with the aid of the newspapers, working in its behalf. Their livers have to get along without it now, and it is difficult to live without the liver; in fact, I do not think it can be done. It has had its day, along with the pile pastes of the past, which indelicate newspapers would sometimes place under a wedding or church announcement or a protracted meeting.

Then there are the commercial tapeworm cures and the peripatetic tapeworm curer, who always keep a tapeworm on hand in the hopper, with a string to it, to show the victim after the remedy with the croton oil has gone through him, and the cancer cure and curer, with whom every, even the most benign symptom, is evidence of malignancy. Then we have the general symptom liar, helped out as usual by the newspapers in making young men miserable, and driving them into hypochondria, melancholia, pyesthenia, etc.

Next comes the youthful indiscretion liar, akin to the lost manhood Ananias and woman's lying friend, who proclaims that only pale pills for pink people will reach the case; also Julia Jenkins' pumpkin juice, proclaimed to bring back the erring husbands to right ways of living and doing.

Another advertisement on billboards and everywhere simply "are you a woman?" seeking to make the impression that all women need medication. This is like the abdominal-supported man who some years ago advised all women to use them and to induce their husbands to wear them on the principle that what was good for the goose was good for the gander. In this connection we might recall the great popularity of somebody's aromatic Scheiden Schnapps, now almost forgotten, and the Juniper berry cures of the past.

In that elder day, when the bloom of verdant youth was on the writer's confiding cheeks and the iniquities of the patent medicine liar had not blanched his face nor caused the loss of his teeth, he used to look with admiration and wonder at the astonishing picture of a burning mountain pouring out healing lava and the galoots of the patent medicine man who had sequestrated all this fiery sanitation, gathering up the healing balm and carrying it not far away, where its wonderful work was shown on man and beast, for it was good for both in the transformation and transfiguration from before and after taking, showing a dejected man and spavined beast in whose appearance life was not worth living, was plainly visible, suddenly changed, after taking, into a lively smiling creature, as if the earth was now more than good enough for him, and even his horse was laughing and getting the laugh on all gullible mankind, have changed since then and the persuasive proprietary has come to contest the palm of these wonder workers in therapeutics for doctors only, and some medical men have supplemented the newspapers and signboards in giving place to some that would be more appropriate in hades. I do not refer to any in my journal, nor in yours, gentlemen, but to the other fellows. Present company is excepted always.

It would be interesting to analyze the patent medicines that are so thoroughly alcoholized that they are characterized as bracers and required to pay a liquor dealer's license, but time does not permit. Booze in this insidious form is so common that the Commissioner of Internal Revenue forbids druggists to sell them without a license. Dr. George Kober gives a list of over 120 of these from Kudros and the Juniper Kidney Cure to William's Kidney Relief. The list may be found in the A. M. A. Propaganda for Reform on page 110. Eye-openers, night caps, and clergymen's booze are among them, though more eloquently named.

One of the greatest problems in therapeutics is to secure the minimum amount of alcohol in medicinal preparations compatible with right solubility of ingredients. The alcohol should be as near an incipient as possible, and its exact proportion to the potent components should be made known for prescribing purposes as is required by the pure food and drug act.

While we have no right to dictate as to the amount of alcohol in any preparation, nor to say what physicians should or should not become acquainted with through our advertising, i. e., while we can not exercise autocratic powers on this subject, we ought to know in order to intelligently discuss the composition of medicines advertised in our pages. We have to submit whatever is offered, if it really has any reasonable claims as a medicine to the intelligent discrimination of our readers, who are supposed to be physicians of discernment. But some of the proprietary promoters are not so conscienceless as the patent medicine man, but, nevertheless, not so cautious and conservative in their business enthusiasm as we would like to have them.

The proprietaries ordinarily have not the ready-made letters for all kinds of disease, such as may be had for a price by any patent medicine proposition, but their enthusiasm is often a little too unbounded and embarrassing, especially as appears in the reading notices sent us based often on the first experience of juvenile doctors eager to get their names in print and to have it known that they have come among us to astonish the world with new achievements in medicine.

The good proprietaries should beware of the patent medicine boosting features, modest in their reading notices and conservative and cautious about their clinical advice. Some of them say too much and are not cautious enough, especially in regard to hypnotics, against their daytime employment.

But the proprietary subject is too extensive and exhausting for our strength at this time and too perplexing. It is just as important that those who look to us for light should find proprietary illumination in our pages as in a dictionary.

It is for them to say if the light shines too feebily or in off color, how much or how little their promoters prevaricate. We can be autocrats or arbiters only in a very limited and reasonable degree. It is our business to show the goods, though we need not constitute ourselves salesmen. Our patrons may be the purchasers, relying on their own judgment as to what they want. We should insist on actual honest formulae as to therapeutic composition, however, if commendation is expected of us and if our patrons expect us to use the weapon they should seek to know the caliber of the gun, the quality of the shot, how far it will carry, and how correctly it will shoot.

It would be better for the profession if more of the proprietaries come from the brains of experienced physicians without secrecy of essential ingredients, as the code enjoins, and protected by a trade mark of competency.

Good and elegantly prepared proprietaries ought to be encouraged; bad ones ought to be discontinued. The little millionaire-aspiring messenger or soda-counter boy who gets up a concoction which has more kill than cure in it ought not to be encouraged to take up the proprietary business, but the real medical chemist who can give the profession something worth while in facility of administration and agreeableness of composition is the profession's benefactor.

This is wrong. Alcohol diluted only 75 per cent should not be sold for indiscriminate use disguised as a medicine, even on a liquor dealer's license. This is as bad as selling paregoric without a physician's prescription, for diluted alcohol is as unsafe as a habit-producing agent with many as the opium which goes into the make-up, in small quantities of the comphorated tincture of opium.

Drink habitues are being made all too rapidly by ordinary processes without the insidious aid of the patent medicine method of manufacture, and it would be wise and humane if we reduced to the minimum the necessary alcohol used in our medicinal tinctures.

DISCUSSION OF DR. HUGHES' PAPER.

PRESIDENT YOUNG: The gentlemen down to discuss this paper are neither of them here. I know of no one so competent to handle this subject as the author of the paper and Dr. T. D. Crothers, whom I will ask to lead in the discussion.

DR. T. D. CROTHERS: There is a psychology about this subject which makes it very interesting. The great proprietary men have, as a rule, been found to be degenerates. A large number of them have died in insane asylums. The conditions causing their peculiar mentality have existed long before. A man who projects a scheme which has for its foundation falsehood is mentally unsound. There is a series of degenerating influences, beginning with brain feeding and ending with brain paralysis, that lead to this condition.

Another curious thing that has come up recently—the cocaine, morphine and hyoscine dope sellers. It is an enterprise that has developed within the last few years. There are men who go around selling this dope to men of high standing who use the stuff and do not want it known. There is a degeneracy in this and a moral insanity. The public is watching them and detectives are looking for them. They are becoming quite common now, and most of their patrons are in the higher walks of life. A friend of mine, high up in the social world, has had several letters offering to meet him here or there and supply him.

The wilder the statements the more degenerate the man, and the nearer his end. If you follow them up carefully, those who have come into public notice and seemed to fade away, you find a striking similarity in them all—a uniform course from beginning to end, and, if they don't die from their particular disease, they leave a fortune that destroys the family.

A—— made a large fortune, and both father and son died in an insane asylum. Half a dozen men who have come into public attention have made a fortune and died insane or paralytic. When I see a new patent medicine come into sudden prominence and see the large claims made for it and the extensive advertising that is done, I generally suspect something of the sort.

DR. GEORGE F. BUTLER: I wish that I were charitable enough to believe with Dr. Crothers that all men who deceive were degenerates and liable to end their days in an insane asylum. If that were so, there would not be insane asylums enough to hold them. I think it is the same lack of personal honor among patent medicine manufacturers that we sometimes find among doctors and business men—a desire to accumulate a lot of money by any method that presents.

It is true there are not as many patent medicines sold as formerly, but there are just as many people going off after strange gods as ever. Nowadays, however, they take the form of osteopathy, new thought, and a dozen other cults that promise so much and deliver so little.

I believe it is the duty of medical editors and every doctor to stand in the community for personal honor and honesty, and try to be a part of the community, and not limit their entire attention to their profession, to stand out and take part in the education of the people and check, if possible, this tendency to quackery (for it is quackery just as much as the old kind was) and the making of laws that handicap the doctor. Perhaps we have more influence than we know if we would exert it. It behooves a medical editor to stand firmly for what is right and try to educate the people, and there is a great demand for high-class health journals for the people—not new thought, Christian Scientists and that like, but good, practical scientific journals for educated people, and if medical societies would appoint men to give lectures to the people much good would be accomplished. They should be taught the fallacy of these things and the danger of them. We have a great work to perform in this direction, and the longer it is delayed the greater effort it will take.

I don't think these people are all degenerates. They are just almighty anxious to make money by hook or by crook.

DR. W. C. ABBOTT: The history of medicine of the day that Dr. Hughes so well knows has been ably portrayed by him. Dr. Hughes reminds me a little of the Quaker who, sitting by his fireside one night, said, "Maria, all the world is wrong but me and thee, and sometimes I think thou art a little peculiar."

Now, the demands upon the medical profession have produced slowly and surely, but with increasing rapidity, an evolution from these days of which Dr. Hughes speaks. It should be an encouragement to us to hear a paper like this, knowing that we can push forward and attain just as great advancement in the next decade.

The public have a right to know the truth of these things, and, knowing the truth, they will take nothing out of the unknown. Let the first plank of this platform be education. We are too ready to bottle up. We are bottled up for a purpose, and quacks and those who have something to gain are taking advantage of our silence. You have a duty to perform, as editors. Will you neglect it? No. What do the people know about these things? What basis for judgment have they? Are you going to keep still all your lives? Little less terrible than the white-slave traffic is the traffic which you know is being driven along all the time, and we say nothing.

Here is the outlook for you: First demand knowledge for yourself, then give it to your people, and through them to the general public. To them you owe the duty, and no one can pay the price for you. Unless you see and do your duty you are remiss as medical editors and men.

Dr. C. H. Hughes (closing remarks): I am glad to see the interest taken in the discussion of a paper so imperfectly prepared. The points I wish to make (I did not make them very clearly) are that it is our duty to discriminate between the good and the bad and the indifferent. It is the duty of the proprietary man to give us a fair statement of the composition—not necessarily the process, that is his for the benefit of his business, but its composition. Another point, well brought out by Dr. Abbott, is the disposition of the medical profession to hold back and say nothing. That is not as it should be. We ought to enlighten the public on what pertains to their welfare. We are probably the most to blame because they are ignorant of the harm of the patent medicine business. The proprietary medicine that is really something good and new (and many of them are), and brings to light something for investigation, should be encouraged. But for the little soda fountain clerk to dope out some sort of formula and supply it to the public is an insult to the profession.

When the public get to know what they are entitled to know in regard to sanitation matters the reign of isms and pathies will have passed. We will not find the characters to which I have alluded in my paper. We will find people inquiring what is in a bottle of medicine before they buy it. They will go to the physician for advice, and the physician will give them true advice and instruct them properly on these matters.

Another mistake we have made is in making light of the good proprietaries. We have neglected them and turned them over to the little soda fountain boy, and he went into the business, found the benefits in it, and has made a fortune out of it that should have gone to the profession.

A man could talk all day on this subject and still not have exhausted the subject. A doctor who has seen the rise and fall of so many different medicines that have possessed the minds of the people knows something of the power that is in them. I have seen some of them when I was a boy, watched them go out of existence, and then seen them spring up again later on.

We should meet the emergency and have a correct understanding of what these things are, what is good and proper and what is not. When we do that we will encourage the good and discourage the bad.

There is nothing to be gained by ignoring them. There is a growing unrest among educated people, and people are resorting to all sorts of things instead of sending for the doctor, as they used to do; and, unless this is to go on, we must educate them so they can tell the false from the true.

